# A Longitudinal Study for the Empirical Validation of an Etiopathogenetic Model of Internet Addiction in Adolescence Based on Early Emotion Regulation

**DOI:** 10.1155/2018/4038541

**Published:** 2018-03-07

**Authors:** Silvia Cimino, Luca Cerniglia

**Affiliations:** ^1^Department of Dynamic and Clinical Psychology, Faculty of Medicine and Psychology, Sapienza University of Rome, Rome, Italy; ^2^Uninettuno Telematic International University, Rome, Italy

## Abstract

Several etiopathogenetic models have been conceptualized for the onset of Internet Addiction (IA). However, no study had evaluated the possible predictive effect of early emotion regulation strategies on the development of IA in adolescence. In a sample of *N* = 142 adolescents with Internet Addiction, this twelve-year longitudinal study aimed at verifying whether and how emotion regulation strategies (self-focused versus other-focused) at two years of age were predictive of school-age children's internalizing/externalizing symptoms, which in turn fostered Internet Addiction (compulsive use of the Web versus distressed use) in adolescence. Our results confirmed our hypotheses demonstrating that early emotion regulation has an impact on the emotional-behavioral functioning in middle childhood (8 years of age), which in turn has an influence on the onset of IA in adolescence. Moreover, our results showed a strong, direct statistical link between the characteristics of emotion regulation strategies in infancy and IA in adolescence. These results indicate that a common root of unbalanced emotion regulation could lead to two different manifestations of Internet Addiction in youths and could be useful in the assessment and treatment of adolescents with IA.

## 1. Introduction

In the last decade, several studies depending on different theoretical frameworks have focused on Internet Addiction (IA) in adolescence [1–3]. The availability of new media and the ever-growing number of youths engaging worldwide in online activities have brought to the fore the urgent need in research for the individuation of their potential negative consequences and of the factors predicting over- or misuse of the Web. The clinical features of behavioral problems related to Internet use have been labeled in several different ways, including* Internet Addiction disorder* [4],* pathological Internet use* [5],* specific or generalized pathological Internet use* [6], and* problematic Internet use* [7]. Despite not recognizing it as an actual diagnosis, the American Psychiatric Association (APA) has recently indicated the* Internet Use Disorder* (IUD) as a clinical condition worth further studies, in the fifth edition of the Diagnostic and Statistical Manual for Mental Disorders [8], proposing it as a form of addictive disorder, with the following criteria: (a) a loss of control over the behavior, (b) conflict (internal and interpersonal), (c) preoccupation with the Internet, (d) using the Internet to modify mood, and (e) withdrawal symptoms [9].

### 1.1. Etiopathogenetic Models

Several etiopathogenetic models have been conceptualized for the onset of Internet Addiction [10]. Scholars focusing on neurobiology and neuroimaging studies have posited that adolescents are at risk of developing IA due to their incomplete neurobiological maturation resulting in their only partially effective cognitive control [11] and reduced boundary setting skills [12]. Some authors have demonstrated a diminished inhibitory control [13] connected with an altered activity in the anterior and posterior cingulate cortices [14] and a reduced frontostriatal top-down control. Because of these neurobiological alterations, adolescents could fail in moderating their use of the Internet experimenting an overwhelming impulse to indulge in online activities (which, on the other hand, are posited to highly activate the reward circuitry [15]). With regard to the functionality of neurobiological connections in adolescents' brains, it has been suggested that youths with IA show a reduced functional connectivity in the circuits joining cortical and subcortical regions, which regulate patterns and behavioral schemes and whose malfunction could lead to a tendency to act as a limited repertory of activities, potentially leading to rituals and maladaptive habits (such as misusing the Web). Other authors have proposed a “component model,” that is based on symptoms and encompasses neurobiological and psychosocial risk factors [16] or a neuroendocrinological model [6, 17, 18]. Yet another branch of literature, mostly in the attachment theory framework [19, 20], has suggested that stressful or traumatic experiences in the first years of life, dissociative mechanisms, and preexisting psychopathology could be at the basis of adolescents' IA [21, 22]. Finally, a model incorporating a neurobiological and cognitive-behavioral approach [6] proposed that Internet Addiction could be fostered by distal or proximal causes. The former incorporates preexisting psychopathology (e.g., anxiety, depression, and substance dependence), whereas the latter relates to individual maladaptive cognitions, so that the misuse of the Web could represent a compensatory strategy to cope with unpleasant feelings of anxiety, depression, loneliness, inadequacy, or frustration [2].

### 1.2. The Present Study

The present study aimed to fill a gap in the literature, adopting a Dyadic-Systemic framework and referring to the concept of* early* emotion regulation, which to our best knowledge has never been used as etiopathogenetic hypothesis for Internet Addiction in adolescents (although it has been used to account for substance dependence [23]). The Dyadic-Systemic model focuses on intersubjective interactions between the child and his caregiver and on the impact of the quality of his affect regulation processes on the development, from childhood to adolescence and adulthood [24]. In fact, affect regulation has been defined as “the process of initiating, maintaining, modulating, or changing the occurrence, intensity, or duration of internal feeling states and emotion-related physiological processes […] often achieved through effortful management of attention […] and cognitions that affect the interpretation of situations […] as well as through neurophysiological processes” [25]. Schore posited that “the core of the self lies in patterns of affect regulation that integrate a sense of self across state transitions, thereby allowing for a continuity of inner experience” [26]. Conventionally, research has observed mother-child interactions through video-recorded sessions, registering spontaneous and mutual contingency between them and reciprocal regulation of dyadic emotional communication [27–29]. According to this framework, mothers interpret child's emotional signals and adjust their affective states providing stimulation, while regulating the intensity of offspring arousal. On their part, children synchronize with their mothers' affects being reactive to their stimulation and reacting contingently to maternal emotions (this concept has been defined as* mutual interactive regulation; *[30–32]).

In general, the quality of mother-child interactions has been posited as the key precursor of children's ability to regulate their inner affective states later in life. While this approach has the merit of extracting one, easily interpreting predictive index of adaptive or maladaptive child's development (the quality of emotional-behavioral exchanges), it nonetheless brings the risk of overlooking the possible predominance of either self-regulation or other based regulation processes in the child. In spontaneous dyadic interactions, in fact, these two factors are both continuously active, but the* balance* between them may shift, due to specific characteristics of the partners of the dyadic systems. For example, an individual characteristic of the child (e.g., difficult temperament) can negatively influence the maternal capacity of interpreting and consistently responding to offspring signals or requests by* interacting with* psychopathological symptoms in the mother (e.g., depressive or withdrawal symptoms).

The adaptive balance between self-regulation and other regulation processes determines the formation of the self, the personal sense of self-efficacy and self-esteem, and above all the possibility for the subject to form and maintain intimate bonds [32]. Moreover, according to this model, this equilibrium prepares to the possibility for the individual of coping with loneliness and negative feelings [33]. Conversely, an excessive other based regulatory action in the child may lead to an internalized state of hypervigilance, whereas predominance of self-regulation (due to a lack of the caregiver's interactive sensitivity) may orientate the child towards the opposite extreme of withdrawal and inhibition. Previous literature has already suggested that both in adolescence and in adulthood an impaired capacity of regulating negative affects could contribute to the onset of Internet Addiction. On the other hand it is sensible to hypothesize that emotion regulation processes in youths and adults may be predicted by emotion regulation strategies in childhood [34]. Thus, in this study we took into account the children's behavioral strategies operated at two years of age aimed at reducing negative feelings and their possible links to IA in adolescence.

## 2. Conceptual Model and Hypotheses

In particular, in this study we examined whether and how emotion regulation strategies (self-focused versus other-focused) at two years of age were predictive of children's internalizing/externalizing symptoms measured when youths were eight years old, which in turn fostered Internet Addiction (compulsive use of the Web versus distressed use) at fourteen. We hypothesized two main maladaptive pathways, so that Hypothesis 1 was as follows: self-focused strategies tended to predict internalizing children's symptoms, which in turn predicts distressed use of the web through Hypothesis 2, which was as follows: other-focused strategies tended to predict externalizing children's symptoms, which in turn predict compulsive use of the Web.

**Figure 1:**
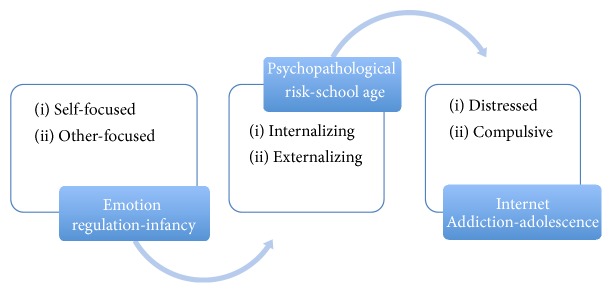
Conceptual model.

The proposed conceptual model is shown in Figure 1.

## 3. Participants and Procedure

The study is part of a larger research that has been carried over thanks to the collaboration of public mental health centers in Italy. The study was approved by the ethics committee of the Psychology Faculty at Sapienza, University of Rome (number: 2005-12), before the start of the study, and was in accordance with the Declaration of Helsinki. These centers had been operating on the territory since 2005 (and they are still operating) implementing prevention and intervention programs for the general population. A longitudinal protocol over three assessment points (2, 8, and 14 years of age of the child) is applied to the families attending these programs and it includes the video-recorded observation of parent-infant interaction in the first years of life to assess children's affect regulation strategies and the screening of mothers, fathers, and children's psychopathological risk at three waves of follow-up. This protocol is intended for the identification of at risk families (due to maladaptive interactive exchanges and/or psychopathological risk either of the child or the parents), so that the clinical services typically propose psychological and/or pharmacological intervention to the families.

In 2016 a group of *N* = 142 adolescents (mean age = 13.8; SD = 2.3) suffering from Internet Addiction without comorbidity were selected from the above population and included in this study. Video-recordings and psychometric measures belonging to families with adolescents with IA were selected from the database and considered for this study. All youths in this clinical population who had been diagnosed for IA without comorbidity were included in this study (in the general sample comorbidities were ADHD, 6.2%; social anxiety disorder, 5.3%; obsessive compulsive personality disorder, 9%; borderline personality disorder, 3%; eating disorders, 4.2%; gambling, 2.6%; and antisocial disorders, 4.7%); families with parents with a referred psychiatric diagnosis were excluded. The families recruited for this study were not included in any treatment plan (because they refused to pursuit one or could not follow one due to geographical distance from the mental health centers) but they had an adolescent with an identified Internet Addiction. Data from parental psychopathological risk and from father-child interactions in this subsample of youths with IA were not available or they were incomplete. Thus, in the present study we considered only data coming from mothers and parental psychopathological risk was not considered. Starting in 2005, families were visited at their homes when children were 2, 8, and 14 years old (T1; T2; T3). At T1, based on previous research [35–37], toddlers' behavioral strategies for emotion regulation were observed during 8 minutes parent-child interactions; at T2, mothers were given the Child Behavior Checklist (version 6-18; [38]); at T3, a clinical interview was conducted with youths based on the criteria of Beard and Wolf [39] to diagnose Internet Addiction and the Generalized Problematic Internet Use Scale 2 (GPIUS2; [40]).

### 3.1. Measures

#### 3.1.1. Early Emotion Regulation

At T1, based on previous research [35], toddlers' behavioral strategies for emotion regulation were observed during 8 minutes parent-child interactions. Mothers were instructed to capture the attention of their offspring with a toy (unfamiliar to the child) and to begin a play interaction. After five minutes, however, mothers and fathers were told to cease the interaction and start reading a paper. This was intended to create a state of mild distress in the children, so that their affect regulation strategies could be observed. After three minutes, the mothers were instructed to cease reading the paper and soothe the child, if needed. If the child was too upset, the recording was stopped and/or never initiated.

Toddlers' self-focused or other-focused strategies were identified and used for the scoring. The scoring was realized including key children and mothers' events, as defined in the literature for mother-infant interactions [41, 42]. Therefore, only clearly discernable and discrete behaviors were considered (smiles, grimace, emission of words or sounds, cry, self-other directed or reciprocal touch, and intentional and purpose-directed movements in the room). All video-recordings were watched by clinical psychologists, blind to the aim of this study, to verify whether the children showed distress cues. According to experts, all children showed signs of distress. No recording needed to be interrupted due to excessive distress of the child. Toddlers' behaviors were scored on a presence/absence basis. Coders were trained psychologists, experts of the field. Toddlers' other-focused strategies included looking at the mother, gesturing to the mother, and talking to the mother. Self-focused strategies included visual distraction (looking away from the parent and/or from the toy), talking to self, self-soothing behaviors, and holding the toy without playing with it. After scoring the strategies, the coders assigned the video-recording to one of the following categories on the basis of the mean of child's observed behaviors: predominantly self-focused; predominantly parent-focused; balanced (with an equilibrium between self- and parent-focused strategies).

#### 3.1.2. Internalizing/Externalizing Symptoms

At T2 mothers were administered the Child Behavior Checklist, version 6-18 (CBCL; [38]) (Italian validated version; [43]). It is a self-administered questionnaire containing 118 items. Parents respond to the items on a three-point scale (0 = not true, as far as you know, 1 = somewhat or sometimes true, or 2 = very true or often true) based on the past 6 months. The measure taps eight empirically based syndromes (anxious/depressed, withdrawn/depressed, somatic complaints, social problems, thought problems, attention problems, rule-breaking behavior, and aggressive behavior) and three broad-band scales (internalizing, externalizing, and total problems). In this study, Cronbach's alpha value was *α* ≥ 0.88 and we considered the two main syndrome scales for internalizing/externalizing problems.

#### 3.1.3. Internet Addiction

At T3 IA was detected by the psychologists and psychiatrists operating in the mental health service on the basis of the criteria proposed by Beard and Wolf [39]. All the following criteria (1–5) must be fulfilled and the subject must (1) be preoccupied with the Internet (thinking about previous online activity or anticipating the next online session); (2) need to use the Internet with increased amounts of time in order to achieve satisfaction; (3) have made unsuccessful efforts to control, cut back, or stop Internet use; (4) be restless, moody, depressed, or irritable when attempting to cut down or stop Internet use; (5) have stayed online longer than originally intended. Moreover, at least one of the following issues must be present and the subject must (1) have jeopardized or risked the loss of a significant relationship, job, and educational or career opportunity because of the Internet; (2) have lied to family members, therapist, or others to conceal the extent of involvement with the Internet; (3) have used the Internet as a way of escaping from problems or of relieving a dysphoric mood (e.g., feelings of helplessness, guilt, anxiety, and depression). Furthermore, adolescents were administered the Generalized Problematic Internet Use Scale 2 (GPIUS2 [40]), which is a revised and updated version of the 15-item Generalized Problematic Internet Use Scale [44]. The GPIUS2 taps four core components: (1) POSI (i.e., preference for online interaction; example item: “Online social interaction is more comfortable for me than face-to-face interaction”); (2) Mood Regulation (example item: “I have used the Internet to make myself feel better when I was down”); (3) Deficient Self-Regulation (example items, resp.: “I find it difficult to control my Internet use”); and (4) Negative Outcomes (example item: “I have missed social engagements or activities because of my Internet use”). For the aims of this study and on the basis of Kandell [45] and Hirschman [46] the four dimensions were aggregated into two scales: distressed use of the Web (incorporating dimensions 1 and 2) and compulsive use of the Web (incorporating dimensions 3 and 4).

## 4. Results

Descriptive statistics for all variables are presented in Table 1.

**Figure 2:**
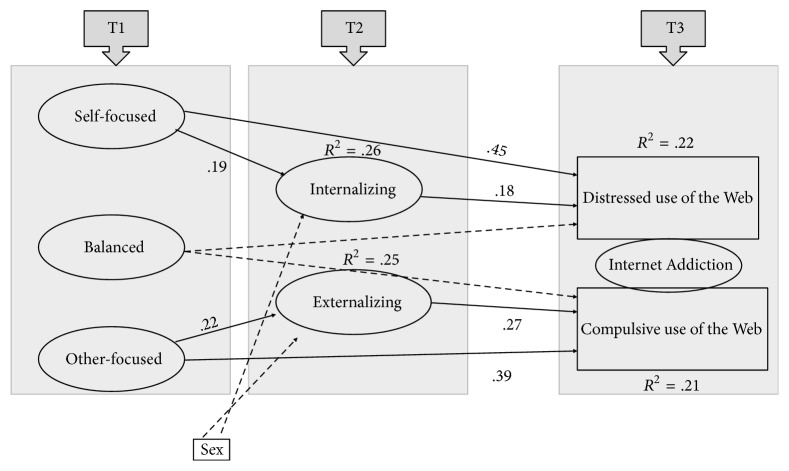
Longitudinal model.

To validate our hypotheses the theoretical model presented in Figure 2 was tested using a latent variable framework.

Self-focused and other-focused emotion regulation strategies were defined as latent variables by using their indicators to account for measurement error. Due to the number of items in the measurement instruments in relation to the number of subjects, all other variables were posited as a single indicator latent variable following Bollen and Long [47] recommendation. To account for measurement error in these cases and to obtain more precise estimates of structural parameters, error variance for each single indicator was fixed at one minus the sample reliability estimate of the variable, multiplied by its sample variance. Mplus 6.12 was used to test this model.

After examination of the Modification Indices, the hypothesized model was corrected to incorporate a direct effect from emotion regulation strategies (self-focused and other-focused) to Internet Addiction (distressed and compulsive). The revised model (Figure 2) provided an excellent fit to the data as revealed by the fit indexes: *χ*^2^(57) = 71.131, *p* = .18; CFI = .94; TLI = .98, RMSEA = .000 (CI = .000–.0681), *p* = .84; SRMR = .072. As shown in Figure 2, results of this model confirmed our hypotheses but it also showed that self-focused and other-focused emotion regulation strategies (unbalanced strategies) directly predicted, respectively, distressed and compulsive Internet Addiction. Moreover, balanced emotion regulation strategies showed a negative association with IA. With regard to Hypothesis 1, self-focused strategies predicted internalizing children's symptoms, which in turn predicted distressed use of the Web; with regard to Hypothesis 2, other-focused strategies predicted externalizing children's symptoms, which in turn predicted compulsive use of the Web. Overall, predictors explained 26% of the variance in internalizing, 25% of the variance in externalizing, 22% of distressed IA, and 21% of compulsive IA.

## 5. Discussion

In a sample of adolescents with Internet Addiction, this longitudinal study aimed at verifying whether and how emotion regulation strategies (self-focused versus other-focused) at two years of age were predictive of children's internalizing/externalizing symptoms measured when youths were eight years old, which in turn fostered Internet Addiction (compulsive use of the Web versus distressed use) at fourteen. Our hypotheses were that two main maladaptive pathways would be present. One would characterize adolescents who showed self-focused strategies of emotion regulation when they were two years old and subsequently presented internalizing symptoms at eight years of age; at fourteen years of age, these individuals had been hypothesized to show a distressed subtype of Internet Addiction. The other maladaptive pathway would differentiate youths who showed other-focused strategies of emotion regulation when they were two years old and successively presented externalizing symptoms at eight years of age; at fourteen years of age, these individuals had been hypothesized to show a compulsive subtype of Internet Addiction.

Although a vast literature has posited that many factors can contribute to Internet Addiction, including individuals' difficulty in coping with stress and developmental challenges [48–50] and controlling social anxiety [51–53] and escapism from unpleasant feelings which cannot be controlled [53–55], no study to our best knowledge has specifically addressed the central role of early emotion regulation, measured as early as in the first two years of life. Actually, emotion regulation has been considered in this field but limitedly to the weight of concurrent ER in adolescents or adults, whereas no attention has been given to the predictive power of this variable when measured in early childhood. Similarly, while several studies have investigated the role of concurrent psychopathological risk in individuals with IA, only a few scholars have granted attention to the possible predictive power of psychological problems for the onset of this disorder and they did not consider psychopathological symptoms in school-age children, concentrating instead on adolescents. This is surprising, given that emotion regulation is increasingly being incorporated into models of psychopathology [56] and great number of studies in the field of developmental psychopathology addressed the negative outcomes of emotion dysregulation in infancy on emotional-behavioral functioning in children and on its subsequent links with other clinical conditions (e.g., depression, anxiety, substance addiction, and attachment insecurity) [57, 58]. It must be said, however, that IA is a relatively novel clinical manifestation and this branch of research will be able to count on results from new longitudinal studies.

In this study, results confirmed our hypotheses demonstrating that early emotion regulation has an impact on the emotional-behavioral functioning in middle childhood (8 years of age), which in turn has an influence on the onset of IA in adolescence. Moreover, our results showed a strong, direct statistical link between the characteristics of emotion regulation strategies in infancy and IA in adolescence. In our sample, infants who predominantly used self-focused strategies to regulate negative emotions were at higher risk of developing a distressed IA subtype, whereas young children who primarily used other-focused strategies to adjust to negative emotions were at higher risk of developing a compulsive IA subtype. These results are consistent with the literature in the field of emotion regulation, as defined by authors considering its relational component (rather than its relations with individual characteristics). In this view, excessive self-focused regulation in infancy can be related to withdrawal symptoms later in life [30] and impaired emotion regulation in general can be associated with a difficulty in structuring and maintaining intimate relationships with others. It can also deepen the impact of a negative view of self-embedded in the insecure internal working models in adolescence and foster difficulties in identifying and describing feeling [59], which in turn can predict the overuse of the Web to seek virtual contacts and technology-mediated interactions that can be perceived as easier to control. On the other hand, preponderant other-focused ER strategies in infancy are proposed to foster an emotional hyperactivation that can be also defined as a hypervigilance [60, 61]. It is known that one of the issues characterizing IA is the fear of missing out, that is, the preoccupation of being left out of interesting, important, or relevant circles of information and relationships. In this view, our results suggest that this preoccupation could be related to an early unbalance in the emotion regulation strategies, so that the subject has not learned to rely on himself/herself to regulate negative feelings, using others instead for this aim.

From a psychodynamic standpoint, these two configurations (distressed and compulsive Internet Addiction) are two faces of the same coin and they serve the same psychological need. The distressed subject (formerly a self-focused child with internalizing symptoms) develops an addiction to the Internet in the attempt to emotionally interact with others (against his/her withdrawal tendency). The subject who uses the Internet in a compulsive way, instead, seeks comfort in the obsessive contact with virtual others to strengthen and/or define his self-identity, which had not fully developed due to the continuously other-oriented early emotion regulation strategies. This conceptualization is derived from Hirschman's theory [46] that posited that a person susceptible to addiction might fall into two subtypes: distressed and sociopathic. Distressed subjects preferably use an external locus of control (due to a sense of self-doubt, incompetence, and personal inadequacy) and this tendency results in being easily subjective to environmental factors (e.g., addictions). Sociopathic subjects, instead, have sensation-seeking tendencies and experience the need for immediate sensory gratification. In such a conceptualization, both types of subjects use the addiction to conserve a stable sense of self. We propose that the same mechanism operates in subjects with Internet Addiction when they showed unbalanced early emotion regulation strategies. The approach indicating two main types of Internet addicted adolescents could be useful in the assessment and treatment of youths with this clinical manifestation; on one hand, a clinician could encounter subjects who are apparently well adapted to the environment, have several friends, have good academic results, and are still overusing the Web. We propose that these adolescents could have experienced an unbalance in their early emotion regulation and could have stably used other-oriented regulations. Therefore, they could compulsively use the Internet in an attempt to experience a sense of self-regulation (which had been lacking in their childhood). This hypothesis is in line with Schimmenti and Caretti's seminal work [59]. These authors posited that a deficit in emotional self-regulation might lead to a dangerous use of dissociative mechanisms, activated as to defensively exclude painful mental states, eventually predicting the withdrawal into technological addictions.

On the other hand, other subjects with IA could appear as isolated and withdrawn; these adolescents might have suffered an unbalance in their emotion regulation strategies in the sense of a stably self-oriented regulation, and they could overuse the Web to seek an external means of regulation, which they lacked in their childhood. In both cases, adolescents could over- or misuse the Internet in an attempt to repair the unbalanced emotion regulation strategies.

This study has some limitations. First, we did not assess parental psychopathological risks, which have been widely indicated as key predictive factors for the development of children and adolescents' maladaptive psychological functioning. In particular, we could not include data on children's emotion regulation strategies observed during father-child interactions (although they are included in the original protocol), due to incomplete or unavailable data. Furthermore, the homogeneity of the sample, in terms of cultural, geographical, and socioeconomic status, limits replication of the study in other countries or cultures.

Notwithstanding these limitations, this study can have useful clinical implications in that it may help recognize the “core” underpinning mechanism of IA in adolescents (impaired emotion regulation) while planning prevention and intervention programs based on the specific clinical manifestations of the patients, which can be different. Some youths can show positive symptoms, have many friends, and have apparently good adaptation to the environment but still manifest a difficulty in regulating their use of the Web (and of social network in particular, as these allow them a continuous control over peers); some others can rather show negative symptoms, have a few or none significant relationship with peers, and have poor academic results, tending to isolate themselves and fall into technological* psychic retreats* [59, 62], which they use as shelters against unpleasant feelings. In both cases, however, clinicians should consider very carefully the deconstruction of such strategies, because they can account for a defensive attempt to compensate weakness of the adolescent self. Therefore, preventive or therapeutic programs based on abrupt prohibition of using the Internet (or radically reducing its utilization) could cause an emotional breakdown.

## Figures and Tables

**Table 1:** Descriptive statistics and correlation matrix.

	M	SD	2	3	4	5	6	7
(1) Self-focused ER	.39	.11	.12	.11	.42^*∗∗*^	.09	.61^*∗∗*^	.13^*∗*^
(2) Other focused ER	.45	.15	-	.09	.13^*∗*^	.39	.08	.59^*∗∗*^
(3) Balanced ER	.21	.18		-	.12	.06	.08	.05
(4) Inter. symptoms	25.3	2.9			-	.14^*∗*^	.43^*∗∗*^	.11
(5) Extern. symptoms	22.1	3.1				-	.12	.57^*∗∗*^
(6) Distressed IA	4.2	1.6					-	.12
(7) Compulsive IA	3.98	1.2						-

^*∗*^ *p* < .05. ^*∗∗*^*p* < .001.
